# Comparison of precision of a paperless electronic input method versus the conventional paper form in an andrology laboratory: a prospective study

**DOI:** 10.1186/s12610-024-00248-9

**Published:** 2025-01-13

**Authors:** Kevin K. W. Lam, Percy C. K. Tsang, Connie C. Y. Chan, Evans P. K. Ng, Tak-Ming Cheung, Raymond H. W. Li, Ernest H. Y. Ng, William S. B. Yeung

**Affiliations:** 1 Department of Obstetrics and Gynaecology, School of Clinical Medicine, Li Ka Shing Faculty of Medicine, The University of Hong Kong, Pokfulam Road, Hong Kong SAR, Hong Kong; 2 Department of Obstetrics and Gynaecology, Queen Mary Hospital, Pokfulam Road, Hong Kong SAR, Hong Kong

**Keywords:** Semen analysis, Andrology, Data precision, Data entry, Analyse du Sperme, Andrologie, Précision des Données, Saisie des Données

## Abstract

**Background:**

Manual counting for semen analysis is recommended by the World Health Organization. Technicians performing this usually record their results on a paper worksheet and then enter the data into an electronic laboratory information system. One disadvantage of this approach is the chance of post-analytical transcription errors, which can be reduced by checking the computer entries before reporting by another technician. Such practice inevitably increases the running cost and delays the reporting time. The present study was to establish a paperless electronic data entry system for semen analysis and compare its precision with the conventional paper method.

During semen analysis, readings on the cell counter were video recorded. The precision of the paper record entries was determined by comparing them with the corresponding video records. Patient characteristics and semen analysis results were input directly into an in-house developed data entry system via a tablet computer immediately after analysis. The same set of data was also handwritten on a paper form and was subsequently input into a standard computerized database according to routine practice. The agreement of the data entries between the two systems was then compared.

**Results:**

A total of 787 semen analyses were included in the study, involving 201 samples in Phase I and 586 samples in Phase II of the study. Phase I was the initial learning period. The overall rate of transcription error of the paper form was 0.07%, whereas that of the paperless system was 0.17%. In phase II, the paperless system was modified according to users’ comments. The transcription error rate of the paper form was 0.05%, while that of the paperless system was substantially reduced to 0.01% (*p* = 0.008).

**Conclusion:**

The paperless system is a reliable tool for recording data from semen analysis compared with the conventional paper form. However, training is needed to reduce the error rate of the paperless system.

## Résumé

**Contexte:**

Le comptage manuel pour analyse du sperme est recommandé par l’Organisation Mondiale de la Santé. Les techniciens qui effectuent cette opération enregistrent généralement leurs résultats sur un formulaire papier, puis saisissent les données dans un système d’information électronique de laboratoire. L’un des inconvénients de cette approche est le risque d’erreurs de transcription post-analytiques, qui peuvent être réduites en vérifiant les entrées de l’ordinateur avant de les signaler par un autre technicien. Une telle pratique augmente inévitablement les coûts de fonctionnement et retarde le délai de compte-rendu. La présente étude visait à établir un système de saisie électronique sans papier pour l’analyse du sperme, et à comparer sa précision avec la méthode conventionnelle sur papier.

Au cours de l’analyse du sperme, les lectures sur le compteur cellulaire ont été enregistrées sur vidéo. La précision des entrées des enregistrements papier a été déterminée en les comparant avec les enregistrements vidéo correspondants. Les caractéristiques du patient et les résultats de l’analyse du sperme ont été saisis directement dans un système de saisie de données développé en interne via une tablette immédiatement après l’analyse. Le même ensemble de données a également été écrit à la main sur un formulaire papier et a ensuite été saisi dans une base de données informatisée standard conformément à la pratique courante. La concordance des saisies de données entre les deux systèmes a ensuite été comparée.

**Résultats:**

Au total, 787 analyses de sperme ont été incluses dans l’étude, impliquant 201 échantillons dans la phase I et 586 échantillons dans la phase II de l’étude. La phase I a constitué la période d’apprentissage initiale. Le taux global d’erreur de transcription du formulaire papier était de 0,07 %, tandis que celui du système sans papier était de 0,17 %.Lors de la phase II, le système sans papier a été modifié en fonction des commentaires des utilisateurs. Le taux d’erreur de transcription du formulaire papier était de 0,05 %, tandis que celui du système sans papier a été considérablement réduit à 0,01 % (*p* = 0,008).

**Conclusions:**

Le système sans papier est un outil fiable pour l’enregistrement des données d’analyse du sperme par rapport au format papier conventionnel. Cependant, une formation est nécessaire pour réduire le taux d’erreur du système sans papier.

## Background

Electronic interfaces are reliable and convenient methods for transferring analytical data from an instrument to any laboratory information system (LIS). Owing to the technical nature of semen analysis and resource limitations, andrology laboratories performing manual semen analysis [1] are mainly reliant on manual data entry for reporting results. The semen parameters are first recorded on a paper worksheet and the data are subsequently entered manually into the LIS.

Manual data capture is inexpensive and easy but has several disadvantages, including but not limited to, time-consuming transcription of data, handwriting recognition problems and incorrect data entry. Among these, the post-analytical transcription error is a risk in data reporting. The error rate depends on the nature of the data entered. The transcription error rate ranges from 0.83% to 5% in a clinical laboratory depending on whether it is a numerical or text entry [2, 3].

Understandably, the main reason for transcription error is the precision of data entry. The error of manual entry has been studied in several clinical research settings. For instance, in a study that analyzed patient-recorded outcome questionnaires, the manual error rate was 1.01% for double-digit entries and 2.02% for single-digit entries [4]. In another study comparing manual input data with electronically imported data in a urology clinic, the overall error rate was 2.8%, but individual field errors could be as high as 6.4%, especially for entries in text format [5].

In our andrology laboratory, semen analysis is performed manually according to recommendations of the World Health Organization (WHO) [6]. The raw data are first handwritten on a paper worksheet. These data are then input into an in-house developed database for storage and results reporting. To ensure the precision of the data transfer, the manually input data are cross checked against the paper worksheet by another technician. This procedure is labour-intensive and time-consuming and prone to error. To save manpower, a paperless electronic data report system involving a handheld tablet computer was developed. The aims of the current study were: 1) to investigate the feasibility of establishing a paperless data entry system for an andrology laboratory; 2) to compare the agreement of data entered directly into the electronic data report system and the current method of handwritten data on a paper worksheet followed by entry to the LIS.

## Materials and methods

The study was conducted in a university-affiliated andrology laboratory in Hong Kong. The laboratory can handle over 1,500 semen samples annually. The study complied with local data protection regulations.

### Participants

Semen samples submitted for pre-marital and fertility check-ups between May 2020 and Feb 2021 were used in the study. All the samples submitted for semen analyses were included.

### Study design

The study was conducted with a prospective, parallel-group design. Patients submitted their semen samples together with a semen submission form. Upon sample receipt, the laboratory technician recorded the time received and assigned a sample number on the semen submission form. For every sample received, the relevant patient information and semen analysis parameters were documented in parallel by writing on a paper worksheet by one technician (paper group) and entering an electronic form by another technician (paperless group). Two technicians were assigned to participate in this study and were rotated between the two groups. The study was conducted in two separate periods. After the first period, the electronic forms were amended for better workflow according to the feedback from the technicians.

A paperless electronic data entry system was constructed from the scripting language, Hypertext Preprocessor (PHP). It utilized a web-based interface, in which the electronic entry forms were accessed through the internet via a handheld tablet computer. Patients’ characteristics and semen parameter values were stored in a designated server. Only registered tablet computers could access the database via a designated IP address to protect data privacy. Technicians were required to log in to the data entry system with their usernames and passwords for all electronic input procedures.

For the paper group, patients’ information was handwritten on the paper worksheet by transcribing the information from the semen submission form. Macroscopic examination and semen analysis were performed manually according to routine procedures [6] by using an upright microscope (Olympus, Tokyo, Japan). Sperm motility, sperm count, sperm vitality and sperm morphology were recorded by a laboratory electronic differential tally counter (Modulus Data System, Redwood City, USA). The sperm count was assessed by haemocytometer with improved Neubauer ruling (HBG, Hessen, Germany). The monitor of the counter was recorded by a video-capturing device (Samsung, Suwon, South Korea) throughout. After the analysis, all information on the paper worksheet was input manually into the LIS. For the paperless group, the patients’ information on the semen submission form was scanned into a web-based electronic form using a handheld scanner (Scanmarker, Kansas City, USA) that could detect text in the scanned images and convert them into electronic input to the tablet computer (Microsoft, Redmond, USA), and the semen analysis parameter values on the electronic differential tally counter were entered directly into the electronic form by using a handheld computer (Microsoft, Redmond, USA). The data entered were transferred directly to a separate database for data storage.

The results of the two systems were compared for any discrepancy. The semen submission forms and the videos captured were considered the gold standard for the patients’ characteristics and semen analysis results, respectively. Data transcription or data entry was defined as incorrect if the data in the computer did not match the information captured on either of these two media. As the study only involved additional data entry steps, ethical approval and patient consent had not been sought. No human subject was recruited.

### Statistical analysis

Data were analyzed by the IBM SPSS software (SPSS 26.0, IBM Corporation, USA). Nominal data were expressed as frequencies and percentages. The two study groups were compared with the Chi-squared test for categorical variables. A *P*-value of < 0.05 was considered statistically significant.

## Results

**Fig. 1:**
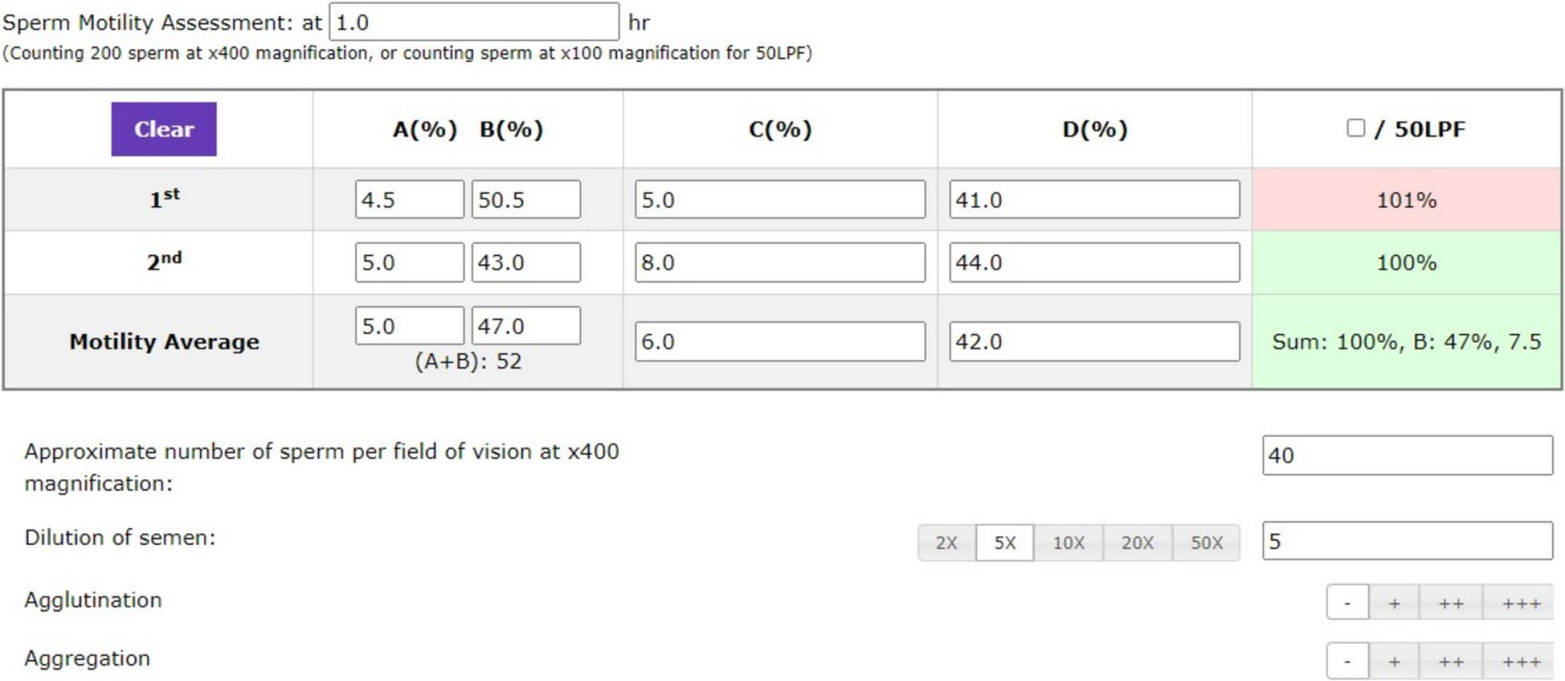
Screenshot of the electronic data input interface. The entry fields for sperm motility assessment are shown. A warning signal (in red) was shown on the second row of the last column when the sum of all motility grades was not equal to 100%. At least 400 spermatozoa were assessed in two replicates for motility assessment. The values between the two replicates were within the 5% error limit. Abbreviation: 50LPF – 50 low power field

**Fig. 2:**
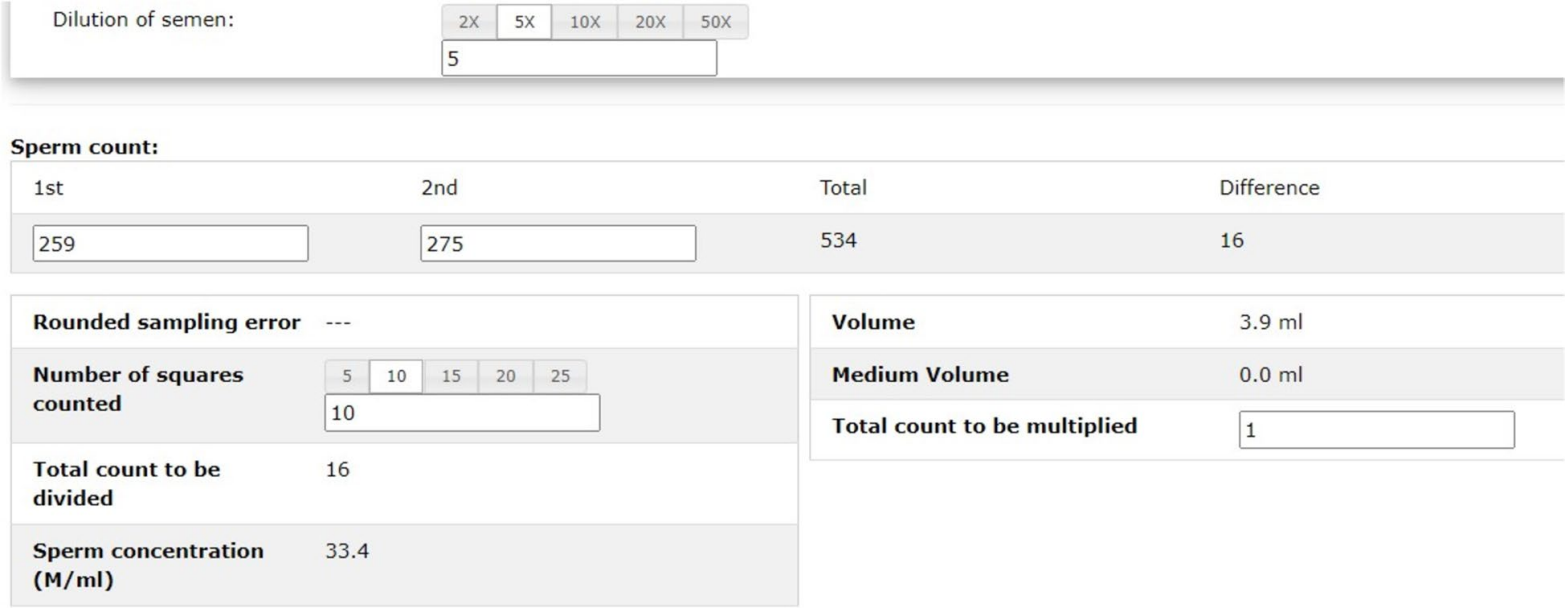
Screenshot of the electronic data input interface. The entry fields for sperm concentration assessment are shown. At least 400 spermatozoa were assessed in two replicates for sperm concentration assessment. The values between the two replicates were within the 5% error limit. Rounded sampling error will be shown when less than 400 spermatozoa were counted

Table 1 summarizes the data and the type of data collected in the paperless group and the paper-based group. Several auto-checking and auto-calculation functions were implemented in the electronic system to reduce input errors. For sperm motility assessments, an auto-checking function was included to check the sum of all motility categories and a warning signal would be generated if the sum was not equal to 100% (Fig. 1) or if the difference between the replicated counts was larger than the accepted limit. The paperless system calculated the concentration automatically from the dilution of the semen, number of grids counted and the number of spermatozoa in the counting chambers, as entered by the technicians (Fig. 2). The system also identified samples via a barcode reader and pre-printed barcode labels. Reports were generated and printed out after data entry.

**Table 1:** Date collected in the paperless group and the paper group

Category of data collected	Data collected	Type of data input	Data collected in paperless group?	Data collected in paper group?
Sample information	Identification number of patient	Numerical	Yes	Yes
	Full name of patient	Text	Yes	Yes
	Date of birth of patient	Numerical	Yes	Yes
	Identification number of spouse	Numerical	Yes	Yes
	Full name of spouse	Text	Yes	Yes
	Date of birth of spouse	Numerical	Yes	Yes
	Date of sample submission	Numerical	Yes	Yes
	Requesting clinic	Text	Yes	Yes
	Assigned sample number	Numerical	No	Yes
	Duration of sexual abstinence	Numerical	Yes	Yes
	Time of sample collection	Numerical	Yes	No
	Time of sample acceptance	Numerical	Yes	No
	Location of sample collection	Categorical	Yes	No
	Sample collection method	Categorical	Yes	No
	Completeness of sample collection	Categorical	Yes	No
	Time receiving the semen sample	Numerical	Yes	No
	Semen submitted by patient or other personnel	Categorical	Yes	No
	Remarks	Text	No	Yes
Macroscopic examination parameters	Liquefaction time	Categorical	Yes	Yes
	pH of the ejaculate	Numerical	Yes	Yes
	Colour of the ejaculate	Categorical	Yes	Yes
	Consistency of the ejaculate	Categorical	Yes	Yes
	Volume of the ejaculate	Numerical	Yes	Yes
Microscopic examination parameters	Percentage of progressively motile sperm (first count)	Numerical	Yes^a^	Yes; average of the two counts^a^
	Percentage of progressively motile sperm (second count)	Numerical	Yes^a^	
	Percentage of non-progressively motile sperm (first count)	Numerical	Yes^a^	Yes, average of the two counts^a^
	Percentage of non-progressively motile sperm (second count)	Numerical	Yes^a^	
	Percentage of immotile sperm (first count)	Numerical	Yes^a^	Yes, average of the two counts^a^
	Percentage of immotile sperm (second count)	Numerical	Yes^a^	
	Time of motility analysis post ejaculation	Numerical	Yes	Yes
	Sperm concentration (first count)	Numerical	Yes^b^	Yes, average of the two counts
	Sperm concentration (second count)	Numerical	Yes^b^	
	Percentage of sperm with normal morphology	Numerical	Yes	Yes
	Percentage of viable sperm	Numerical	Yes	Yes

This table compares the data collected in the paperless and the paper group, with the data categorized into numerical, text or categorical entries

The values between replicate assessments were within the acceptable difference

^a^ = data entry fields with auto-checking function for the sum of all motility grades

^b^ = data entry fields with auto-calculating function for sperm concentration

A total of 787 semen analyses were included in the study, involving 201 samples in Phase I and 586 samples in Phase II of the study. Table 2 summarizes the data composition of the two methods. The number of entries per semen analysis was 23 for the paper group and 32 for the paperless group. The paperless group had 9 more data entries per semen analysis than the paper group. When looking into the type of data, most data were numeric, followed by text and categorial entries. There was no statistical difference among the data entry types between the two groups.

**Table 2:** Summary of data composition in the two groups

	Paper group	Paperless group	*p*-value
Number of entries per semen analysis (N)	23	32	n/a
Percentage of numerical entry (n)	69.6% (16)	68.8% (22)	0.95
Percentage of text entry (n)	17.4% (4)	9.4% (3)	0.38
Percentage of categorical entry (n)	13.0% (3)	21.9% (7)	0.40

This table compares the data composition in the paper and the paperless group

Data were presented as % (n/N) and compared using the Chi-squared test

A *p*-value of < 0.05 was considered statistically significant

The percentages of incorrect entries are summarized in Table 3. In Phase I, there were 3 incorrect entries associated with 4623 entries of the paper group and 11 incorrect entries associated with 6432 entries of the paperless group. No statistically significant difference was detected (0.07% vs 0.17%, *p* = 0.121). All incorrect entries in the paper group were text entries. For the paperless group, more than half of the incorrect entries were numerical entries (54.5%). There were four missed entries in this group accounting for 36.5% of all errors.

**Table 3:** The percentage of incorrect entries in the two groups

	Paper group	Paperless group	*p*-value
**PHASE ONE (201 semen samples)**			
Total number of entry fields (N)	4623	6432	n/a
Percentage of incorrect entries (n)	0.07% (3)	0.17% (11)	0.121
Type of incorrect entries (n / %)	Text (3 / 100%)	Numerical (6 / 54.5%) Missed (4 / 36.4%) Text (1 / 9.1%)	n/a
**PHASE TWO (586 semen samples)**			
Total number of entry fields (N)	13,478	18,752	n/a
Percentage of incorrect entries (n)	0.05% (7)	0.01% (1)	***0.008***
Type of incorrect entries (n / %)	Numerical (3 / 42.9%) Missed (3 / 42.9%) Text (1 / 14.3%)	Text (1 / 100%)	n/a

This table compares the percentage of incorrect entries in the paper and the paperless group

The table is subdivided into phase one (initial learning period) and phase two (post-learning period)

Data were presented as % (n/N) and compared using the Chi-squared test

A *p*-value of < 0.05 was considered statistically significant

In Phase II of the study, the percentage of incorrect entries was similar to that of Phase I for the paper group (0.05%). However, there was a marked reduction in incorrect entries for the paperless group. The number of incorrect entries was reduced to only one. This was a typographical error in a patient’s name. There was a significant difference in the percentage of incorrect entries between the two groups (0.05% vs 0.01%, *p* = 0.008).

## Discussion

Electronic interfaces are the most accurate method for transferring data from laboratory instruments to the LIS for data recording and results reporting. Many laboratory tests, including point-of-care tests, depend on manual entry because of technical barriers and the nature of the tests, which do not allow direct transfer of test data between the instruments and the LIS. According to the guidelines of the latest WHO laboratory manual for the examination and processing of human semen [1], the manual method is the method of choice for performing semen analysis, which is being used by most andrology laboratories in Hong Kong [6], however, there is no recommendation on the method of data entry. Here, we reported the establishment of a tailor-made paperless electronic input system for better data integrity for semen analysis in the andrology laboratory.

Our paperless input system has several advantages. First, the results of the semen analysis are directly entered into the LIS. This eliminates the need for a second data entry step from the paper worksheet into the LIS and for time-consuming data checking with the conventional paper method. Second, it reduces transcription errors due to recognition problems of the handwritten paper worksheet. Third, electronic scanning is used to reduce the potentially high error rate in transcribing text information of patients into the LIS [5]. The future use of a barcode patient identification system could further reduce transcription errors and keep the manpower needed at the minimum level.

The electronic forms are specially designed to reduce human input errors by auto-checking and auto-calculation of the data. With the feedback from the technicians, several tailor-made functions are included in the system. For instance, the sum of the sperm motility of all grades should add up to 100%. The averages of the motility parameters and the sperm concentrations are calculated automatically from the replicates. If the difference between the two counts is larger than the accepted limit, the user will be alerted. Any blank field that requires a compulsory entry will be highlighted before the record is saved. In case of extreme values of sperm morphology (> 10% normal), pH of the ejaculates (pH value < 7.0) and ejaculate volume (> 7.0 mL) encountered, the technicians will be warned to confirm the correctness of the input data.

There was a marked difference in the number of entry fields between the two groups. One of the reasons was that replicated counts in sperm motility and sperm concentration assessments were entered in the paperless group for the calculation of average values. In the paper group, only the averaged values were entered. Secondly, the dilution factors used and the number of grids counted in the haemocytometers during sperm concentration assessment were entered into the paperless group. However, only the calculated sperm concentrations were included in the paper group. Lastly, uncommon scenarios including incomplete ejaculation or submission of semen samples by personnel other than the patient were all documented in a single, free-text remark entry field in the paper group. These scenarios were listed as separate categorical (yes or no) inputs in the paperless group.

In Phase I of the study, all incorrect entries were text entries in the paper group, i.e. transcriptional errors from reading the handwriting on the paper worksheets, involving incorrect input of patients’ names and the requesting clinic’s code in the LIS. For the paperless electronic input group, numerical input errors and missed entries constituted the majority of the errors. These incorrect entries may happen when the technicians in the initial stage were unfamiliar with the electronic input methods, the virtual keyboard layouts and the design of the electronic forms.

In Phase II of the study, the percentage of incorrect entries remained low and was similar to that of Phase I in the paper group and understandable, as the participating technicians were well trained for this traditional data entry method. For the paperless group, there was only one incorrect entry found in Phase II of the study. This low rate is supported by data from a randomized study that compared the efficiency of data entry between electronic and paper reports in clinical research, in which no data entry error was reported with the electronic report form [7]. The marked reduction in the data entry error rate in the paperless group could partly be contributed by adequate training in Phase I and consequent familiarization of the technicians with the electronic input method, but the possibility of the technicians being overly cautious during data input cannot be ruled out. Another prospective study concluded that the efficiency of electronic data input could be enhanced by 30–50% after three weeks of training [8]. In the present scenario, a training of 200 semen analyses should be sufficient to train a technician owing to the relatively simple data entry format as most entry fields are numerical entries.

Apart from training, quality control activities should be carried out to ensure the reliability of the data input. Regular data audits should be performed in the andrology laboratory. The monitor of the cell counter is video-recorded to document the counts performed by one technician, followed by verifying the data input in the paperless system by another technician. The tolerance rate of the data input error can be set at 1%. The audit will be performed again for the same technician if he/she falls below the tolerance rate. Retraining on data entry should be considered if the technician has poor performance. The frequency of the audit should depend on the workload of the individual andrology laboratory and the number of staff involved in data entry.

### Limitations of the study

A limitation of this study is that the time-saving effect of the electronic input method against the traditional data entry method was not measured. A few studies have investigated the time saving of electronic input methods; one randomized controlled study reported around 20% of the time was saved by the electronic input method and an average of 5 manpower minutes were saved per electronic report form, owing to data transcription redundancy [7]. Although direct comparison with these studies is not feasible owing to the difference in data complexity, all studies suggest a marked reduction in overall data entry time in the electronic input group.

## Conclusion

Although there was a significant difference in the precision rate between the two groups, both data entry methods attained 99.9% (paper method) to 100% (paperless method) data entry precision rate if we rounded off the figures. In real-world scenario, the difference was negligible. This study provides evidence that the precision of paperless electronic input forms is at least as good as the traditional paper-based data entry method in an andrology laboratory setting. The use of electronic input methods is preferred for better manpower utilization and data quality. The inclusion of auto-checking and auto-calculation functions can reduce human error. However, sufficient training of the technicians is needed before its routine use in clinical practice.

## Data Availability

No datasets were generated or analysed during the current study.

## Abbreviations

LIS: Laboratory information system
WHO: World Health Organization
PHP: Hypertext Preprocessor
